# Family support services delivered using a restorative approach: A framework for relationship and strengths‐based whole‐family practice

**DOI:** 10.1111/cfs.12636

**Published:** 2019-04-02

**Authors:** Annie Williams

**Affiliations:** ^1^ CASCADE/DECIPHer, School of Social Sciences Cardiff University Cardiff UK

**Keywords:** family support services, relationships, restorative practice, strengths based, whole‐family approach

## Abstract

Family support services, an integral part of many welfare systems across the developed world, have witnessed a growing demand for the use of relationship and strengths‐based whole‐family approaches in the belief that this increases service engagement and effect. Despite this, knowledge that delivering services using such approaches can be challenging and calls for the identification and exploration of methods likely to promote and sustain their use. Restorative approach is an ethos and method centred on building and sustaining positive relationships, which is increasingly being adopted in family and children's services in the United Kingdom. Despite this, the scarcity of research conducted in this area as yet, means little is known of its use and effect in this context. This article draws on empirical data collected in a wider study exploring the efficacy of different family service delivery models to describe the use of restorative approach in family service provision and determine whether its adoption promotes sustained use of strengths and relationship‐based whole‐family approaches when working with families. Furthermore, it explores whether the process incorporates wider evidence‐based methods of change.

## 1 INTRODUCTION

The belief that the state has a responsibility to care for families in need has seen family support services become key elements in social welfare systems in many countries (Dolan, Canavan, & Pinkerton, 2006). Although family support services in the United Kingdom are firmly established, their perceived value has wavered with government policy and associated debate between those who prioritize family support and others focused on child protection regardless of family preservation (Canavan, Pinkerton, & Dolan, 2017; Featherstone, White, & Morris, 2014; Frost & Parton, 2009).

Interest in family support services in the United Kingdom is long established as evidenced by the Children's Act 1989, which sets out authority responsibility to safeguard the welfare of children in need via family support when possible. Attention to developing such services can also be seen post‐1997 in the focus on combating poverty and social exclusion in the United Kingdom (Hills & Stewart, 2005) and associated government policy which aimed to improve family support. In practice, this led to programmes, such as “Sure Start,” that sought to improve the lives of families in disadvantaged areas through networks of professional and community support (Bouchal & Norris, n.d.). Wider commitment could be seen in the work of the Social Exclusion Unit (later Task Force) who played a significant developmental role, not least in advocating relationship, strengths‐based practice (Morris et al., 2008) and arguing that to break established cycles of disadvantage, social service providers must “Think Family” as in the use of whole‐family approaches (Hughes, 2010). Such recommendation gave direction to those involved in U.K. early intervention services as it advocated approaches such as Team Around the Family, a relationship, strengths‐based family‐focused form of practice in which teams of practitioners, families, and community members come together to explore the challenges affecting families, provide support, facilitate change, and ultimately benefit the children or young people involved (Institute Public Care, 2012). Despite this, experience has shown that delivering family support services using such methods is challenging (Institute Public Care, 2012; Morris & Featherstone, 2010); raising questions of whether wider methods can better embed and sustain such practice (Tew, Morris, White, Featherstone, & Fenton, 2016).

Restorative approach is being increasingly adopted by family and children's services in the United Kingdom (Mason, Ferguson, Morris, Munton, & Sen, 2017; Williams & Segrott, 2018; Kay, 2015; Finnis, 2016) based on the underlying theory that repairing harm or resolving problematic situations is best achieved by building or restoring relationships rather than penalizing those involved (Hopkins, 2009; McCluskey et al., 2008; Strang & Braithwaite, 2000). This intention to resolve harms is supported by a set of core principles: collaboration, fairness, voluntary participation, respect, honesty, trust, safety, and nondiscrimination; accessibility (Restorative Justice Council, 2015; Restorative Justice Network, 2003); values which determine the nature of restorative work regardless of the settings it is used in.

Restorative approach is based on restorative justice, a practice used in Western justice systems since its development in the 1970s. Restorative justice was founded on ancient methods (Gavrielides, 2007; Wachtel, 2013) which saw offences and problems arising within communities dealt with in collaborative ways that included all affected (Daly, 2001; Van Ness, 2005; Zehr, 2015). Restorative justice is founded on the principles described and a process, which sees an offender, the victim, and others affected by an injustice take part in discussions revolving around acknowledgement of the crime and harmful effects, and consideration and agreement of how the harms could be addressed and repaired as far as is safe and possible (Zehr, 2002).

Restorative approach has been described as an adaption and application of restorative justice to community/organizational contexts, which differs in that it operates on two levels (Williams & Segrott, 2018; Hopkins, 2004, 2009). At the first, restorative principles are employed in everyday contexts with the intent of improving the environments they operate in. At the second, restorative approach is a process used to address problems as they arise. Although such use mirrors restorative justice, restorative approach differs as it seeks to achieve change by concentrating on the problem rather than the person (Hopkins, 2004, 2009; McCold & Wachtel, 2003). Restorative approach is used in various formats ranging from “informal” adherence to its principles and associated skills that elicit affective language and statements, create empathy, and encourage collaboration to the use of more formal practices of restorative circles, mediation, and full restorative conferences (Costello, Wachtel, & Wachtel, 2010). Within this, the “restorative questions”: What happened? What were you thinking/feeling? Who has been affected & how? What do you need for harm to be repaired? What needs to happen now to make changes? are used to draw out accounts of unwanted situations from multiple perspectives, increase empathy, create motivation to change, and stimulate a solution‐focused consideration of how to do so (Hopkins, 2009).

**Figure 1:**
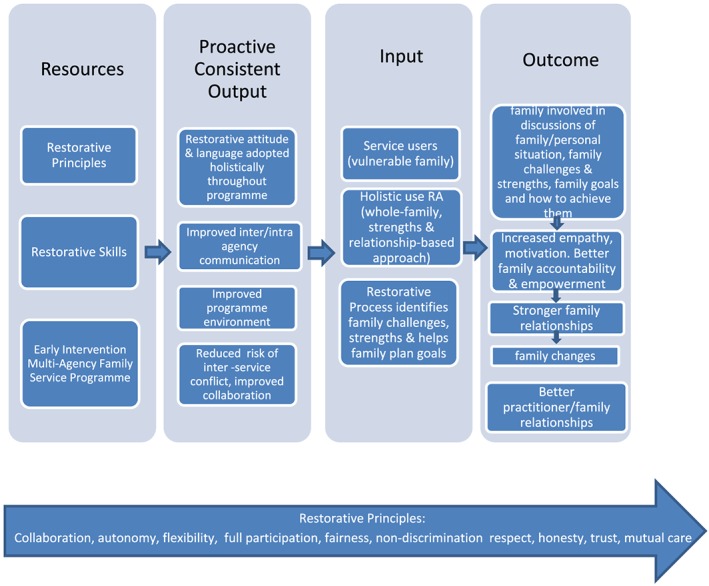
Restorative approach (RA) in family service provision (adapted from Williams & Segrott, 2018) [Colour figure can be viewed at http://wileyonlinelibrary.com]

The question of what restorative approach can offer to family support services has been considered elsewhere (Williams & Segrott, 2018). In this, it is noted that restorative approach has already been successfully employed in disadvantaged communities (Fives, Keenaghan, Canavan, Moran, & Coen, 2013; Lambert, Johnstone, Green, & Shipley, 2011) and children's services (Mason et al., 2017; Tariq, 2016). The authors also observe that the value base of the approach maps well onto relationship, strengths‐based whole‐family practice, as does the associated focus on good communication, mutual understanding of family problems, collaborative discussions of necessary change, and how to achieve them; a process likely to build participant confidence and resilience. A model of restorative approach in family service delivery that links the restorative values and process to relationship, strengths‐based whole‐family services is offered in the article, and a slightly amended version can be found in Figure 1.

The model sets out the potential impact of restorative. Early outputs are changes in organizational culture, environment, and interactions. Later outcomes are dependent upon the provision of family services embedded in restorative approach. Integral to this is the belief that embedding family services in a restorative approach would result in consistent delivery of whole‐family, strengths, relationship‐based approaches.

Despite this argument, whether or not this happens when restorative approach is used in family support services is largely unknown, a reflection of opinion that too little is known of what actually happens when social service providers and users meet (Ferguson, 2014). This article draws on data from a wider study to explore whether the use of restorative approach in family services promotes whole‐family, strengths, and relationship‐based services and furthermore whether the associated process incorporates other evidence‐based methods such as solution‐focused therapy and motivational interviewing.

## 2 METHOD

The wider study explored service delivery in a Welsh national family programme whose guidance calls for the use of relationship, strengths‐based, whole‐family approaches (Welsh Government, 2011). The research conducted included case studies of the family services in a number of local authorities. In one of these, the Team Around the Family (TAF) key‐worker service was provided by an agency long experienced in and committed to the use of restorative approach. Families qualifying for this service had complex needs requiring the involvement of at least four support agencies to address their needs, to be eligible for support.

This article draws on the service delivered by the TAF team to answer the following questions:

- Does a restorative approach framework promote the delivery of whole‐family, strengths, relationship‐based family support services?
- Does the delivery of family services using a restorative framework incorporate wider methods of change?

### 2.1 Data collection

The TAF team were approached by email and in person, given information about the study, and asked to take part in a focus group. The focus group took place in the team office and included eight practitioners, a lead practitioner, and the team manager. Consent was taken before the group, and the discussion was recorded using a digital tape recorder.

After consultation with managers, the TAF team were asked if the researcher could accompany them on family visits. The team proposed that only experienced practitioners took part, and this was agreed. Practitioners obtained consent for the observations from families before visits, confirmation was confirmed by the researcher at the visits, and families were given a voucher as a thank you. Over a period of 3 months, observations of four practitioners making visits to 11 families at different stages of service use (Table 1) took place.

**Table 1:** Stage of service use at observation

Phase	*N*
First meeting	2
Ongoing assessment	3
End of assessment	3
Progress review/case closure	3
Total	11

During visits, observation was structured by the research questions and the theory represented in Figure 1. This directed attention to the restorative principles and how they transferred into practice the process of delivering family services, whether/how using restorative approach led to strengths, relationship‐based whole‐family approaches, and whether the approach generated the use of wider methods of change.

Visits took place across the authority. Each took 45–75 min. Most families were white Welsh; one had recently immigrated. Two visits were conducted with the mother, eight included at least some of the children (14 children in total), three included male partners, and three wider family members.

The researcher attended the visits purely as an observer. The practitioner and researcher met before the visit to allow the practitioner to describe the family background and the purpose of the visit. At the start, the practitioner introduced the researcher to the family. During the visit and with permission from the practitioner and the family, the researcher took extensive field notes.

### 2.2 Data storage and analysis

Focus group data were transcribed ad verbatim, and field notes were written up. Transcripts were stored in university computers in locked rooms protected by university passwords. N‐Vivo 10 software was used to conduct analysis.

Analysis was directed by Figure 1 and focused on understanding of restorative principles, the use of restorative approach in service delivery, how the underlying values shaped practice, and the skills used to convert the approach into practice. Further evidence of strengths and relationship‐based practice and whole‐family approaches and whether this was linked to the restorative values, skills, and process was sought. Overall analysis was focused on

- practitioner perceptions of restorative principles, attitudes, and skills;
- evidence of restorative approach in practice;
- relationships between practitioners and families; and
- commonalities between restorative approach and relationship, strengths‐based, whole‐family practice, and wider behaviour change techniques.

The wider study was given ethical approval by the Social Science Research Ethics Committee, Cardiff University.

## 3 FINDINGS

Findings in the first section are drawn from the focus group, with primary interest in practitioner understanding of restorative approach and perceptions of how it affected practice. Those in the second section are based on family visit observations as well as the focus group with interest in how these beliefs and perceptions translated into practice.

### 3.1 Practitioner perceptions of restorative approach and its impact on service provision

All practitioners had received internally provided restorative training focused on engaging families as well as delivering services. More experienced staff had received additional training from an external agency. Regardless of the training received, the team manager believed all were familiar with restorative approach to the point of being able to run restorative family conferences. When discussing their introduction to restorative approach, a few practitioners described it as innovative: *“a bit of an eye‐opener”* (focus group participant [FGP]), and others felt it brought together an instinctive pattern of person‐centred relationship‐based working:

I was kind of using restorative approach without knowing it. Allowing that person to talk and have their voice heard, trying to understand what they wanted. (FGP)

a description, which echoed family practitioners trained in restorative appaorch elsewhere (Williams, Reed, Rees, & Segrott, 2018). When describing service delivery, practitioners emphasized the importance of communication, describing it as fundamental to restorative practice. When talking of the benefits of good communication, there was agreement that the skill helped build relationships, instigated better understanding of family needs, and gave insight into family strengths. Good communication was seen as essential from the first referral, as building a full picture of the family, and the problems faced is vital when deciding whether families needed the service, met criteria for use, or should be signposted elsewhere.

they need a conversation, they need that like restorative bit of work done, meet with the family have a conversation. We are then able to see where they at in terms of how able they are to meet their own needs. (FGP)

If a family is accepted, a practitioner is assigned to the case and visits the family home. When considering service delivery, some practitioners saw restorative values: being person led, honest, empathetic, partnership based, empowering, non‐judgemental, and democratic (Burford & Hudson, 2000; Lloyd et al., 2007; McCluskey et al., 2008) as central to practice. Others related the values more explicitly to the delivery of relationship‐based practice

it's more about the core principles, about being person‐led and really building a relationship with a person. (FGP)

supporting argument that restorative approach is a relationship‐based method of being and working (Strang & Braithwaite, 2000; McCluskey et al., 2008).

Practitioners also linked restorative approach to strengths‐based practice, notably in association of the approach with partnership‐based practice as recognized in strengths‐based practice literature (Saleebey, 1996; Early & Glenmaye, 2000; Kemp, Marcenko, Lyons, & Kruzich, 2014). A strength‐based approach was also evident in recognition of parental expertise and practitioner willingness to question their own views when they differed from families

they know what's going on, they know how best to keep, (pause) usually the families we work with know how best to keep the kids safe. They know how best to meet their needs. They know how best to manage risk. Sometimes, it gets a bit skewy, but usually they know. Or if we have a different perspective about that, their perspective is still valid. We do a lot of reflection about why they might think a certain way. (FGP)

When describing service delivery, three practitioners supported wider contentions that restorative approach encompasses other strengths‐based methods (Braithwaite, 2011, 2016; Macready, 2009). One practitioner felt that the focus on building mutual understanding of the situation was analogous to motivational interviewing as the practice increased the impetus for change. Another spoke of how practitioners act as “sounding boards” when families reflect on how to achieve change; suggesting the use of solution‐focused therapy. Finally, delivering services using an inclusive whole‐family approach meant spending significant time with families, which gave opportunity to promote social modelling

they start to mirror the way you're working. Parents can see how things are happening and rather than screaming and shouting at their kids, they might kind of think: “Ah I saw how they did it and they got a response and normally I don't get a response.” (FGP)

The employment of a whole‐family approach was further explored by asking what practitioners understood as a “family.” Replies suggested use of a whole‐family approach which sees each family member as a service user (Hughes, 2010).

It could be friends, maybe it's relatives. Whoever they deem as their family unit would be who we would work with … the small child who wants to talk to us, their perspective on what the problems are is as valued as mum's. It may be very different, from a different angle, but nevertheless it's to be heard, respected and integrated into our picture of things. (FGP)

Description of restorative tools that help practitioners translate restorative values into practice was also given. Amongst these, the restorative questions were central and commonly used

that first question, “can you tell me what's happening”, and then moving through, I am always conscious of always moving through that process of what's happening, “how are you thinking or feeling about that, is that having an impact on them, is it having an impact on the wider family, wider community, and what do they need to move forward from that”, and start the planning and look at how they can change and what needs to be in place. (FGP)

with practitioners seeing this as instrumental in encouraging families to communicate and participate in identifying and prioritizing goals rather than accepting professional solutions.

Practitioners with experience of delivering services without a restorative approach compared earlier ways of working negatively

before it's was “ok, so we are going to go in. we are going to find what's going on and then we are going to tell them what, kind of, to do.” [Restorative approach] it's working with the family to explore what's happening so we can come up with the solutions together. (FGP)

With further criticism of time pressures, which had prevented them gaining detailed understanding of families, creating good relationships, and generating feelings of collaborative working

never had 6 weeks, you know, to spend with the family [assessment] … often I've done a 7‐ day assessment and I've gone back and said “this is what you need to do.” It wasn't so much that “what do you need, how can I help you?” I've always had to tell them, so it's different, it's quite different, I didn't like working that way; it's quite controlling. (FGP)

Although practitioners believed that the use of restorative approach was effective in engaging families and facilitating change. They recognized limits. They spoke of the recognized challenge of working with clients against a background of unequal power (Keddell, 2014) and how achieving change in established behaviours can often be slow

we want families to try and make changes but not just like for today, but long term, for the future, and sustain it. And that doesn't come overnight. Whereas statutory services would want you to, you know, “you make that or else the children are gone,” people are just, people can't do that, so it's more working in partnership, more holistically. And more time to do that, and that's the only way you'll be able to get families to make changes because it comes from within, from them. (FGP)

### 3.2 Delivery and receipt of a restorative approach TAF programme

This section draws on the family visit observations as well as the focus group to explore the use of restorative approach in practice.

#### 3.2.1 Assessment

Assessment nominally takes 6 weeks in this agency; in practice, it often takes longer because, as noted elsewhere (Thom, Dalahunty, Harvey, & Ardill, 2014), more time is needed when using whole‐family approaches. Practitioner visits began with general enquiries about the family, reminiscent of the “checking in” of restorative circles (Mirsky, 2007). Enquiries were successful in generating communication and in building relationships as evidenced by progression from limited replies in the first visits to long descriptions of family events later. When describing assessment to families, practitioners used positive language describing it as a “getting to know you” phase. Families were told that the information would be used to write “family stories” from the family perspective. These would be used at the TAF panel meetings and, if families wished, given to other agencies to avoid the continual repetition of stories, an acknowledged barrier to service use (Katz, Corlyon, La Placa, & Hunter, 2007). Having the stories approved by the family was a demonstration of the values of empowerment, honesty, fairness, and democracy central to both restorative (Restorative Justice Council., 2015) and strengths‐based practice (Manola, 2007; Pattoni, 2012; Kemp et al., 2014).

During assessments and other practice observed, positive language was used. This included the use of the restorative questions, often shaped in informal language such as “what's going on?” to gain description of the situation and “how was that for you” to encourage description of linked emotions. Practitioner responses to resultant narratives were marked by active listening: a factor commonly linked to relational, strengths‐based practice (e.g., Early & Glenmaye, 2000; Dunst, Trivet, & Hanby, 2007; Lietz, 2011; Kemp et al., 2014), little interruption, and acceptance of family accounts without reference to counterindicative knowledge. There was liberal praise of family efforts to improve matters, part of a strengths perspective as it increases people's confidence to be producers not recipients (Early & Glenmaye, 2000; Foot & Hopkins, 2010). The impact of this on building good relationships between the practitioner and families was evidenced by two mothers who, at the end of first meetings, said they felt the practitioner was someone they could talk to and could provide previously lacking support.

Family assessments also focused on identifying family needs and goals, with questions like “What do you guys need to make life easier?” used. In two early assessments, practitioners turned the conversation towards addressing needs as they emerged, suggesting the use of “quick gains,” a practice associated with service engagement. When exploring needs, restorative questions were sometimes supplemented by other tools, for example, a card game was used with a child in order to draw out likes, dislikes, and emotions. Although most family conversations were positive, a few saw parents and/or children explaining how proposed resources did not suit them. Methods to address such seeming resistance included conversations about barriers and addressing them, mediation, or suggestions the practitioner give it some thought to discuss in later visits. Such responses demonstrated collaborative, flexible approaches and illustrated the primacy given to respecting family views rather than imposing practitioner decisions.

Evidence of whole‐family approaches was seen widely. One practitioner explained to the family that they worked this way because although one person may be most affected by a problem, the effects impact on the rest of the family. It was also made clear that “family” can include anyone important to them: a neighbour, a friend, and wider and/or immediate family. This advice appeared to have effect as many later observations saw multiple family members taking part in visits. The need for skill and sensitivity when trying to engage more reluctant family members was evidenced in two occasions when male family adults repeatedly walked through the room and responded to practitioner attempts to engage them with short answers before leaving. However, in both cases, the men eventually returned and stayed in the room for longer engagement. When asked about this, one practitioner reflected

they know that you're coming and they've chosen to stick around and sort of be around and doing odd jobs in the vicinity, they can hear everything that's being said and being talked about. And in a way, they're just as engaged in the process because they can still, they're still part of it, they're still part of the engagement even if they're not the ones talking. So you've got this outer layer of people who are around in the house, who are quite significant. I think about it in layers and sometimes I think “well these people have, kind of, at least they've managed to suss me out as a person, at least they've listened to me, kind of, chatting to their mum or they kind of know who I am, and maybe build a little bit of trust. Maybe think: ‘oh you're not a complete' yeah, there's some connection.” ( FGP)

Similar reactions to attempts to engage other family were seen in children: one switched off a television programme as they became involved in the conversation and another was insistent that the practitioner set their 1‐2‐1 meeting before leaving. Further attempts were seen when birth fathers did not live in the house in suggestions that practitioners met and worked with them elsewhere. One mother's negative response led to the issue not being not pressed, but it was not abandoned, and the possibility of involving the birth father in Family Group Conferencing was mentioned later in the visit.

There was further evidence of practitioners meeting family members individually as advocated by restorative approach (McCold & Wachtel, 2003). When proposing meetings with children and young people, parents were asked how they felt about this, and permission was gained before the plan progressed. One later family assessment visit began with the practitioner sharing with parents the positive comments a child had made about family life during a meeting at school.

#### 3.2.2 TAF meeting and goal planning

During assessment, forthcoming TAF meetings were referred to as a “getting together” of all involved: with the purpose of formulating a plan to meet family needs and decide who would do what and why explained. Through a restorative lens, this could be describe as a circle meeting conducted for collaborative decision‐making Coates, Umbreit, and Vos (2003). When talking about proposed meetings, practitioners demonstrated adherence to a whole‐family approach as they placed great importance on the family taking part. As noted earlier, the family “story” is central during the TAF meeting. Although it was explained that this can be edited by the family before use, observation suggested that boundaries exist. For example, having read the story, one parent was concerned that their cannabis use had been documented. The practitioner reassured that the recovery from use was also recorded but did not talk of removing the detail.

#### 3.2.3 Progress review and service monitoring

After the TAF meeting, a phase that saw families use wider services and resources began. During this, practitioners talking of *“stepping back”*, but their visits still seemed important to families; not just to monitor and coordinate services but as a support in its own right.

Review and late assessment visits still featured restorative questions but now used to explore emerging difficulties, service use, and progress. The continued use of questions to explore experiences, related feelings, and intentions reinforced the impression that service use embedded in restorative approach is a collaborative process.

One observation demonstrated much of this. The visit began with the mother wondering if the family needed all services suggested as, with practitioner support, she had “kicked a few demons into touch” and given up substance use. As a result, many problems had been addressed, and “bad” family days were uncommon as family life now felt normal. The practitioner listened, praised the strengths displayed, and commented on how well she had sorted herself out. The practitioner and mother then collaboratively reviewed the situation for each family member. Family conflict had decreased, so the Family Group Conferencing referral was cancelled. One daughter had not yet accessed the careers service as encouraged, so it was agreed this should be completed. When the daughter joined the meeting, she was consulted, and a plan to achieve this was made. They then talked about the child whose situation had triggered service use who was now “pulling sickies,” at his pupil referral unit. On entry, the child was included in the conversation and asked how life and school were generally. The practitioner learnt life was good, grades had improved, and the child had earned enough points to use a punch bag, which had been enjoyable. Enquiries discovered that good behaviour earned points that accessed the punch bag. This led to agreement that truancy would be avoided to gain more points and ensure consistent access to this activity. It was then agreed that the family case would be closed once the careers advice was gained. This anecdote illustrated many elements of working restoratively. Good communication and the development of good relationships was promoted by the practitioner eliciting and listening carefully to family narratives and praising positive progress. This included younger family members being part of the conversation and listened to. When problems needed resolving, conversations engendered a feeling of working it out collaboratively. The work around the child's school attendance gave evidence of restorative approach encompassing motivational practice.

The use of other behaviour change techniques was apparent elsewhere. Solution‐focused therapy was seen in one discussion of how to address a young persons' refusal to use mental health services. First, both practitioner and mum agreed on the service necessity. They then considered how to make this happen. Some practitioner suggestions were rejected by the mother, and the practitioner mused “what can we do?” Mother suggested the youth worker took her son as he hated his mother picking him up from school and she would meet them at the clinic. The practitioner applauded the idea, and they decided to try it out. Social modelling was also observed, not only in the positive language and active listening employed throughout but also when a mother complained of a child never leaving her alone and not liking to play with toys. The practitioner did not respond immediately but later, although engaged in a different conversation, sat on the floor and played with toys. The child came over, engaged, and remained there playing for the rest of the visit.

#### 3.2.4 Case closure

As exemplified above, case closure was negotiated, discussed, and decided in light of family progress and ongoing service demands.

**FP1** it does depend on family, but then we've got to be aware of referrals that are coming in. And it's a balance I think, because we never want to leave a family at a point where things aren't going [well] and they're working with you to make those changes. It's just case by case. **FP2:** It's what they recognise as being ok, rather than what we recognise as being Ok as well. **FP3**: But there's also a tension between that and targets as well. [agreement] there's a, sometimes there's a direct conflict in terms of meeting our targets, etc. And so targets can skew things, maybe make things move along faster or be closed as resolved when actually the practitioner could do with more time. (FGP)

Variation in attitudes to case closure was seen. Unlike the mother above who suggested finishing service use, two were reluctant to finish working even though progress had been made and additional services accessed. In one of these, the practitioner decided to stay a little longer as some additional services had either not been offered or offered in a way the family could not use. In the final case, the mother agreed that she did not need the practitioner as apart from housing, necessary support was in place, and positives have taken over from negatives. Although the mother was reluctant to lose the support, it was no surprise; indeed, multiple instances of practitioners reminding families of the time‐limited nature of the service were seen. This case was closed, but all families were told they could keep the TAF contact and use them if things deteriorated.

## 4 DISCUSSION

This article considered whether using restorative approach promoted whole‐family, strengths, and relationship‐based family approaches in family service delivery.

The definition of “family” given and the efforts made by practitioners to identify and include family members suggested that a whole‐family approach fits well into the inclusive principles surrounding a restorative approach. Observation of practitioners encouraging but not pressurizing reluctant individuals into service involvement gives further illustration of a whole‐family approach and adherence to the restorative principle of voluntary inclusion (Restorative Justice Council, 2015). The high consultation with family children was notable as the opinion and perspectives from younger individuals are often overlooked (Aubrey & Dahl, 2006). There was also evidence of practitioners working at the family level, first in the encouragement of families to contribute to discussions and also in mediation or suggestions or the use of family group conferencing when conflict was present.

Although clear definition of relationship‐based practice is difficult to find, values such as commitment, communication, honesty, and empathy, which are associated with the approach (Munro, 2011; Scott, 2013), were articulated by practitioners and seen in practice. This was especially evident in the communication skills used and the consistency of active listening. This practice, together with the collaboration seen between practitioners and families when considering family challenges or case closure, children taking active part in family visits, and clients saying they could work with practitioners at an early stage, implied good relationships existed or were being built despite the power imbalance still inevitably evident. Overall, the evidence supports definitions of restorative approach as a way of resolving problems by building relationships rather than penalizing those involved (Hopkins, 2009; McCluskey et al., 2008; Strang & Braithwaite, 2000).

When considering whether a restorative approach generates strengths‐based practice, the principles deemed to underlie restorative practice, and the questions used to translate these into practice can be found in the examples given. Many factors evident in these examples: a listening approach, positive praise, identifying family strengths, and encouraging family choice echo those found in the literature concerned with strengths‐based practice (e.g., Saleebey, 1996; Lietz, 2011; Kemp et al., 2014; Canavan et al., 2017).

With such evidence, a key question becomes whether restorative approach adds anything to the use of strengths, relationships, and whole‐family approaches without it. Study findings suggest that consistent use of the values intrinsic to restorative approach forms a good base for relationship‐based practice. In addition, the discovery that the restorative framework provided by the questions and the process is seen as a reference point and model for practice mirrors opinion voiced family practitioners undergoing restorative approach training elsewhere who described the restorative approach process as much needed structure in which to locate practice (Williams et al., 2018). Finally, findings indicate that restorative approach incorporates elements of motivational interviewing, solution‐focused therapy, and social modelling as noted in discussions of restorative practice in other service settings (Braithwaite, 2011, Braithwaite, 2016; Macready, 2009).

Such findings turn attention to other strengths‐based models of social care practice gaining popularity, particularly Signs of Safety (Turnell & Edwards, 1999) and Reclaiming Social Work (Cross, Hubbard, & Munro, 2010). The growing adoption of these models indicates increasing support for relationship, strengths‐based practice in contrast to more traditional punitive, risk‐adverse approaches. However, the existence of seemingly similar models with shared intent but as yet little evidence of effect calls for work to identify and compare these models, the practice they produce, and related efficacy. Potentially, such work could inform whether one model should be preferred, whether applicability varies with context, or whether a single model combining all effective elements of practice is called for. The need for this body of work is strengthened by the varying costs of the training for different methods, especially in the current economic climate, which sees many services adversely affected by financial constraints (Laid, Morris, & Archard, 2017).

Study findings must be considered with knowledge of its limitations. Family visits were negotiated by the TAF team, and it is possible that observations were only of families more positive about and better engaged in services. The sole involvement of experienced practitioners in observations was unfortunate as less experienced practitioners may have practiced differently. The small number of observations is an issue, especially as there was little experience of witnessing work with more resistant families or those at immediate risk of referral to children's services. Finally, the article did not compare the efficacy of using restorative approach in family services with the provision of whole‐family, relationship, and strengths‐based practice without. This question will be addressed in forthcoming work.

## 5 CONCLUSION

The study explored the use of restorative approach in family support service provision, an unrepresented area of interest in U.K. research and wider. Practitioner understanding of restorative approach and direct observation of their family work suggests that using restorative approach as a practice framework engenders whole‐family, relationship, and strength‐based practice and can lead to the use of the wider change methods of motivational interviewing, solution‐focused therapy, and social modelling as part of the process. The study also found that using a restorative approach was acceptable to families most of whom saw the service as a positive help, suggesting it is likely to engage rather than alienate service users. Finally, the article identified connections and similarities between restorative approach and other strength‐based models of practice and asked questions about relative cost and effect, an issue little considered to date.
